# Heterologous Expression of a VioA Variant Activates Cryptic Compounds in a Marine-Derived *Brevibacterium* Strain

**DOI:** 10.3390/md16060191

**Published:** 2018-06-02

**Authors:** Xiao Han, Lukuan Hou, Jing Hou, Yongyu Zhang, Huayue Li, Wenli Li

**Affiliations:** 1 Key Laboratory of Marine Drugs, Ministry of Education, School of Medicine and Pharmacy, Ocean University of China, Qingdao 266003, China; hx131629@163.com (X.H.); houlukuan1991@163.com (L.H.); 17864275159@163.com (J.H.); 2 Laboratory for Marine Drugs and Bioproducts of Qingdao National Laboratory for Marine Science and Technology, Qingdao 266237, China; 3 Research Center for Marine Biology and Carbon Sequestration, Shandong Provincial Key Laboratory of Energy Genetics, Qingdao Institute of Biomass Energy and Bioprocess Technology, Chinese Academy of Sciences, Qingdao 266003, China; zhangyy@qibebt.ac.cn

**Keywords:** heterologous expression, recombinant, *Brevibacterium*, macrodiolide, antibacterial

## Abstract

A new 14-membered homodimeric macrodiolide, brevidiolide (**3**), along with four known aromatic compounds (**1**, **2**, **4** and **5**) were obtained by heterologous expression of the recombinant plasmid pWLI823 expressing the G231L variant of VioA in the marine-derived *Brevibacterium* sp. 7002-073. The structures of **1**–**5** were elucidated on the basis of LC-MS and 2D NMR spectroscopic analyses. In the evaluation for the antibacterial activities of the compounds against multi-drug resistant (MDR) strains, **5** showed notable growth inhibition against *Staphylococcus aureus* CCARM 3090 and *Klebsiella pneumoniae* ATCC 13883, with a minimum inhibitory concentration (MIC) value of 3.12 µg/mL.

## 1. Introduction

Marine-derived actinomycetes, with their extreme living environment featuring low temperatures and high pressure as well as poor nutrient availability, are considered to have great potential to generate structurally novel and biologically active secondary metabolites [1,2]. However, the actinomycete-derived compounds reported to date are just the tip of an iceberg: a large number of the molecules are cryptic due to the silent genes [3]. Thus, activating the silent genes becomes an important key to entering the locked world of inaccessible compounds generated by marine actinomycetes.

Since the 1980s, a wide range of approaches have been used to activate/modulate silent genes in microbes [4,5], among which cell-cell communication by signaling molecules has attracted increasing attention [6]. Small molecules produced by a microbe may act as signal molecules to regulate gene expression in other microbes depending on the treating concentration [7], thus triggering the production of related compounds in the heterologous hosts.

In our previous study, we identified a series of antibiotic violapyrone derivatives, which are encoded by the type III polyketide synthetase VioA, from deep sea-derived *Streptomyces somaliansis* SCSIO ZH66 [8]. The chemical skeleton of violapyrones is quite similar to reported α-pyrone type photopyrones that serve as signal molecules at low nanomolar concentrations (Figure S1) [9], thus, we assumed that the violapyrones might play a similar function, providing the possibility for their potential of activation/regulation of gene expression in the heterologous hosts.

*Brevibacterium* sp. 7002-073 (GenBankID: KY770501.1) was isolated from the cyanobacterium *Synechococcus* sp. PCC7002 collected off the coast of China in the Yellow Sea near Qingdao. *Brevibacterium* is an obligate aerobic, catalase-positive and spore-free Gram-positive actinomycete. Some *Brevibacterium* strains are very useful in industry. *B. linens* is one of the most important surface bacteria in the cheese-making process due to its role in the surface coloring and its typical flavoring activity [10]. *B. flavum* has been used in the mass production of the essential amino acid L-valine [11]. However, the secondary metabolites from *Brevibacterium* have not been reported much, except for a handful of peptides, fatty acids and glycolipids [12,13,14].

**Figure 1:**
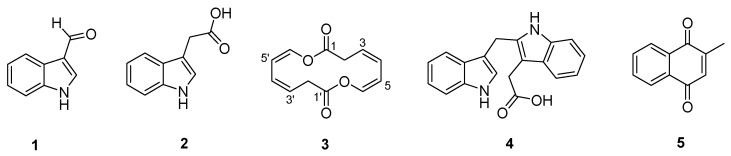
Structures of compounds **1**–**5**.

In this study, to test the possibility of the violapyrones being signal molecules, the recombinant plasmid pWLI823 expressing the G231L variant of VioA (impaired activity, unpublished data), which is a pWLI807 derivative [15], was introduced into *Brevibacterium* sp. 7002-073 by electroporation. In the recombinant strain 7002-073/pWLI823, compound **2** was overproduced 20-fold as compared to in the wild-type strain, and compounds **1** and **3**–**5** were newly accumulated, among which **3** was a new compound with an unusual 14-membered symmetric macrodiolide ring (Figure 1). Herein, we describe the isolation and structure identification of compounds **1**–**5** from the 7002-073/pWLI823 recombinant strain. Moreover, the antibacterial activities of **1**–**5** against multi-drug resistant (MDR) strains were evaluated as well.

## 2. Results and Discussion

To introduce heterologous gene into *Brevibacterium* sp. 7002-073, the genetic system of *Brevibacterium* sp. 7002-073 was firstly established using pMT3 as vector and electroporation as transformation method. Competent cells were prepared as described in the Experimental Section, and then pMT3 (control) and pWLI823 (harboring VioA-G231L) were introduced to generate recombinant strains 7002-073/pMT3 and 7002-073/pWLI823, respectively (Figure S2).

**Figure 2:**
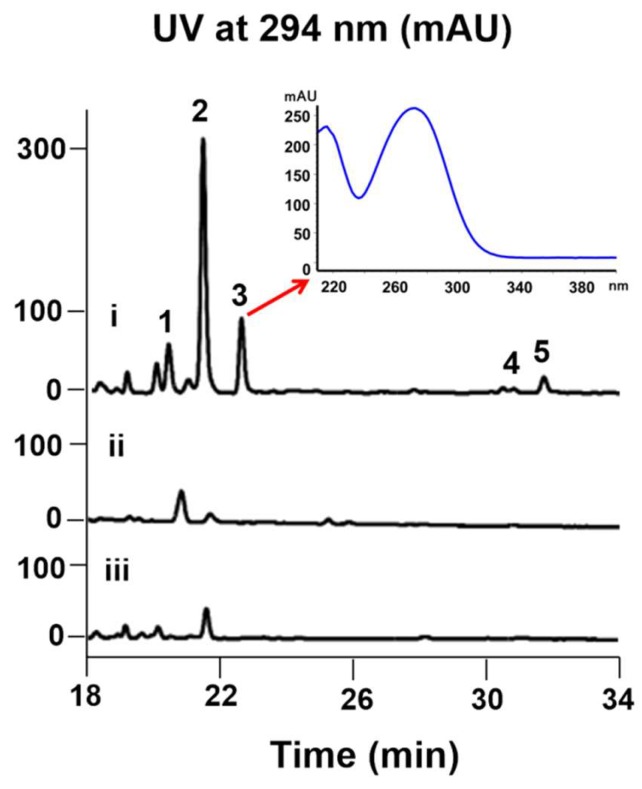
Comparative HPLC analysis of the compound production in the culture extracts of wild-type and recombinant strains of *Brevibacterium* sp. 7002-073; (i) 7002-073/pWLI823; (ii) 7002-073/pMT3; (iii) wild-type 7002-073.

Interestingly, the color of the culture broth of 7002-073/pWLI823 was quite different from that of the wild type, indicating the possible changes in their metabolite production (Figure S3). The fermentation broth of wild-type and recombinant strains (7002-073/pMT3 and 7002-073/pWLI823) of the *Brevibacterium* sp. 7002-073 were extracted with EtOAc. In the HPLC profile of the *Brevibacterium*/pWLI823 strain, we observed that compounds **1** and **3**–**5** were newly generated, and compound **2** was overproduced by 20-fold as compared to the wild-type strain (Figure 2). These results indicated that insertion of the recombinant plasmid pWLI823 led to the compound activation in *Brevibacterium* sp. 7002-073. With the large-scale fermentation of the 7002-073/pWLI823 strain, which was sequentially subjected to EtOAC extraction and reversed-phase chromatographic fractionation as well as HPLC purification, compounds **1**–**5** were obtained.

**Figure 3:**
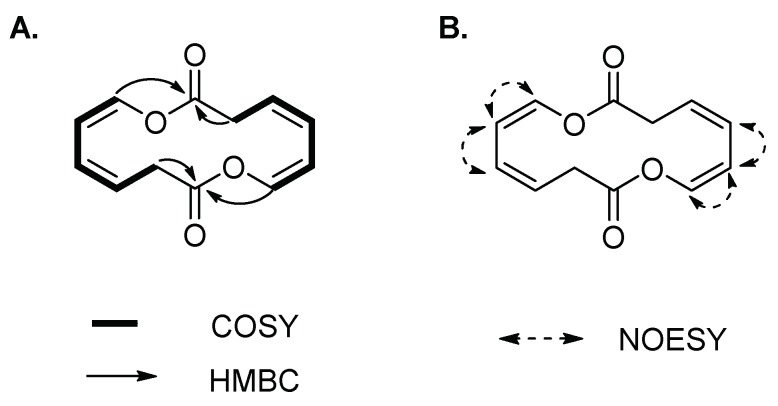
(**A**) ^1^H-^1^H COSY, key HMBC and (**B**) NOE correlations of **3**.

Compound **3** was isolated as a yellowish amorphous solid. The molecular formula of **3** was established as C_12_H_12_O_4_ on the basis of the HR-ESIMS data ([M + HCOOH − H]^−^ at *m*/*z* 265.1467 calcd 265.0712) (Figure S4). The structure of **3** was elucidated by 1D and 2D (COSY, HSQC, HMBC and NOESY) NMR spectroscopic analysis (Figures S5–S9). The ^1^H and HSQC spectra of **3** disclosed a methylene (δ_H_ 3.66) and four olefinic protons (δ_H_ 5.95, 6.04, 5.48 and 6.60), which sequentially comprise a spin system of H-2/H-3/H-4/H-5/H-6 according to the COSY spectrum (Figure 3A). In the HMBC spectrum, except for the corresponding carbon signals of C-2~C-6, only one additional carbonyl carbon C-1 (δ_C_ 167.4) was observed, which showed correlations with H-2, and H-6 (Figure 3A). The conformations of two double bonds were confirmed to be *cis* by combination of the coupling constant values and NOE correlations of H-4/H-5 and H-5/H-6 (Table 1 and Figure 3B). Thus, compound **3** was deduced to be cyclic oxepin-2(3*H*)-one. However, the molecular formula of oxepin-2(3*H*)-one (C_6_H_6_O_2_) does not match with the experimental HR-ESIMS data of **3**, in which each number of atoms was double of the supposed structure, revealing the formation of a symmetric homodimer. Thus, compound **3** was finally identified as a new 14-membered homodimeric macrodiolide, named brevidiolide. This conclusion was further supported by the chemical shift estimation with ChemDraw 15.0. The ^1^H and ^13^C NMR chemical shift values of **3** are shown in Table 1.

Compounds **1**, **2**, **4** and **5** were isolated as yellowish amorphous solids. The HR-ESIMS analysis disclosed that the molecular formulas of **1**, **2**, **4** and **5** were C_9_H_7_NO, C_10_H_9_NO_2_, C_19_H_16_N_2_O_2_ and C_11_H_8_O_2_ with molecular ion peaks at *m*/*z* 146.0598 [M + H]^+^, 176.0706 [M + H]^+^, 303.1132 [M − H]^−^ and 173.1170 [M + H]^+^, respectively (Figures S10, S15, S17 and S22). The structures of **1**, **2**, **4** and **5** were determined by NMR data assignment and comparison with the reported literatures [16,17,18,19], which were identified as indole-3-carboxaldehyde, indole-3-acetic acid, 2-(1*H*-indol-3-ylmethyl)-1*H*-indole-3-acetic acid, and 2-methyl-1,4-naphthalenedione, respectively (Figure 1). The ^1^H and ^13^C NMR chemical shift values of **1**, **2**, **4** and **5** are listed in Tables S1–S4.

The heterologous expression of the recombinant plasmid pWLI823 expressing the G231L variant of VioA in the *Brevibacterium* sp. 7002-073 activated the production of a novel 14-membered macrodiolide (**3**). Macrodiolides are a class of unusual microbe-derived natural products that are grouped into homodimers and heterodimers according to the building block symmetry [20,21,22]. Among the reported macrodiolide structures, the 14-membered ring is rather rare, with only one heterodimeric colletodiol family being discovered from the fungus *Clonostachys cylindrospora* [23]. To the best of our knowledge, compound **3** is the first 14-membered homodimeric macrodiolide obtained from nature. These results indicated the possibility of the violapyrones acting as signal molecules for activation of the silent microdiolide biosynthetic gene cluster in *Brevibacterium* sp. 7002-073.

In our investigation for potent antibacterial activity of compounds **1**–**5** against MDR strains, **5** exhibited notable inhibitions against *Staphylococcus aureus* CCARM 3090 and *Klebsiella pneumoniae* ATCC 13883 with a minimum inhibitory concentration (MIC) value of 3.12 µg/mL. Compounds **1**–**4** showed null inhibition against all of the tested MDR strains up to 12.5 µg/mL (Table 2).

## 3. Materials and Methods

### 3.1. General Experimental Procedures

^1^H, ^13^C, COSY, HSQC, HMBC, and NOESY (mixing time = 142 ms) NMR spectra were recorded on Bruker Avance III 600 spectrometers at 298 K. The mixing time used for the NOESY spectrum was 142 ms. Chemical shifts were reported with reference to the respective solvent peaks and residual solvent peaks (δ_H_ 3.31 and δ_C_ 49.0 for CD_3_OD; δ_H_ 2.50 and δ_C_ 39.5 for DMSO-*d*_6_). HR-ESIMS data were obtained on a Q-TOF Ultima Global GAA076 LC-MS spectrometer. Optical density (OD) measurements of ELISA experiments were recorded on a TECAN infinite M1000 Pro multi-detection microplate reader. High-performance liquid chromatography (HPLC) was performed on an Agillent 1260 Infinity apparatus with a diode array detector (DAD).

### 3.2. Strains, Plasmids, and Culture Conditions

*Brevibacterium* sp. 7002-073 (GenBank accession No: KY770501.1) was isolated from the cyanobacterium *Synechococcus* sp. PCC7002, which was collected off the coast of China in the Yellow Sea near Qingdao, China. The multi-drug resistant (MDR) bacterial strains *Staphylococcus aureus* CCARM 3090, *Escherichia coli* CCARM 1009, *Enterococcus faecalis* CCARM 5172, *Enterococcus faecium* CCARM 5203, and *Salmonella typhimurium* CCARM 8250 were bought from the Culture Collection of Antimicrobial Resistant Microbes (Seoul Women’s University of Korea), and *Acinetobacter baumannii* ATCC 19606 and *Klebsiella pneumoniae* ATCC 13883 were bought from the American Type Culture Collection. The plasmids used in this study are listed in Table S5.

The *Brevibacterium* sp. 7002-073 strain was routinely cultured at 30 °C in the brain heart infusion broth (BHI) liquid medium or on BHI agar plate. BHI medium supplemented with 2% glycine and 10% sodium succinate was used for electro-competent cell preparation. The *Brevibacterium* sp. 7002-073 transformants harboring plasmid were incubated in the BHI medium with thiostrepton (5 µg/mL). The fermentation medium (M8) consists of 2% soluble starch, 1% glucose, 0.2% meat extract, 0.2% yeast extract, 0.3% CaCO_3_ and 0.4% casein (pH = 7.0). The MDR bacterial strains were cultured in Luria-Bertani (LB) medium at 37 °C.

### 3.3. Transformation Procedures

The transformation procedures for *Brevibacterium* sp. 7002-073 was established with reference to the literature [24]. When the OD_570_ reading reached 1.3–1.5, the cell culture was cooled on ice for 10 min, followed by centrifugation at 4 °C, 5000× *g* for 10 min. After washing twice with 20 mL of cold sucrose (0.8 M), the cells were resuspended in 1/100 volume of the same solution and were frozen in liquid nitrogen for 30 s. The competent cells (100 μL) were mixed with 100 ng of plasmid DNA and loaded into a prechilled 2-mm gap electroporation cuvette. After 10 min of incubation on ice, the cell-DNA mixture was shocked by a single 25 kV/cm pulse generated by Bio-Rad Gene Pulser apparatus (Bio-Rad laboratories, USA). The 0.7 mL of recovery medium (BHI medium supplemented with 0.5 M sucrose) was added to the cells right after the pulse delivery. After incubation at 30 °C for 90 min, the cells were then spread onto BHI agar plates supplemented with thiostrepton (5 μg/mL), and incubated at 30 °C for 2 days. Transformants were verified by PCR with the primer pairs listed in Table S6.

### 3.4. Isolation and Purification

The fermentation broth (50 mL) of the *Brevibacterium* sp. 7002-073 in M8 medium was extracted with EtOAc, and was subsequently subjected to the HPLC analysis. Analytical HPLC was performed with a linear gradient from 5% to 80% B/A in 40 min (phase B: 100% ACN + 0.1% HCOOH; phase A: H_2_O + 0.1% HCOOH; YMC-Pack ODS-A column 150 mm × 4.6 mm, i.d. 5 µm; wavelength: 210 nm) to analyze the production changes between the wild-type and recombinant strains. The culture broth (16 L) of 7002-073/pWLI823 was extracted with EtOAc at room temperature, which was partitioned between 90% MeOH and *n*-hexane to remove nonpolar components. Then the MeOH layer was subjected to a stepped-gradient open column (ODS-A, 120 Å, S-30/50 mesh) eluting with 20–100% MeOH to yield 13 fractions. Compounds **1**–**3** (0.39, 8.76 and 0.57 mg, respectively) were obtained from fraction 3 on a reversed-phase HPLC (YMC-Triart C18 column 250 mm × 10 mm, i.d. 5 µm; wavelength: 210 nm) eluting with 70% MeOH + 0.1% HCOOH (*v*/*v*) (1.5 mL/min). Compound **4** (0.85 mg) was obtained from the fraction 5 eluting with 75% MeOH + 0.1% HCOOH (*v*/*v*) (1.5 mL/min). Compound **5** (1.18 mg) was obtained from fraction 6 eluting with 70% MeOH + 0.1% HCOOH (*v*/*v*) (1.5 mL/min).

*Brevidiolide (***3***)*: yellow amorphous solid; UV (MeOH) *λ*_max_ (log ε) 207 (3.75), 236 (3.27), 262 (3.45), 310 (2.58) nm; ^1^H and ^13^C NMR data, see Table 1; HR-ESIMS *m/z* 265.1467 [M + HCOOH − H]^−^ (calcd. for C_12_H_12_O_4_, 220.0736).

### 3.5. Antibacterial Activity Assay

The antibacterial activities of compounds **1**–**5** against MDR bacterial strains were tested by the minimum inhibitory concentration (MIC) method [8]. The MDR strains were seeded in LB medium and then incubated at 37 °C for 18 h. After dilution with LB broth to 10^6^ cfu/mL, the 190 μL of cell suspension was dispensed into 96-well plates. Different concentrations of sample solutions in MeOH were dispensed into 96-well plates. The LB broth was used as a blank, and the methanol and tetracycline were used as a negative and a positive control, respectively. The growth of MDR strains was measured after 18 h of incubation at 37 °C on a microplate reader (Epoch2, Biotech) at the wavelength of 600 nm. Each assay was performed in triplicate.

## 4. Conclusions

In summary, we introduced the recombinant plasmid pWLI823 into the marine-derived *Brevibacterium* sp. 7002-073, leading to accumulation of five compounds (**1**–**5**), among which **3** was a novel compound with an unusual 14-membered homodimeric macrodiolide skeleton. In the test of their potent antibacterial activity against MDR strains, compound **5** exhibited notable inhibition against *S. aureus* and *K. pneumoniai*. These results demonstrated that the current strategy provides new opportunities for the discovery of novel cryptic compounds from marine actinomycetes, and furthermore, it may be used as an efficient tool for antibiotic lead compound digging as well.

## Figures and Tables

**Table 1:** The ^1^H (600 MHz) and ^13^C (150 MHz) NMR chemical shifts of **3** in CD_3_OD.

Position	*δ*_H_ (*J* in Hz)	*δ*_C_
1		167.4
2	3.66, m	29.8
3	5.95, m	127.3
4	6.04, dd (6.6, 9.0)	126.1
5	5.48, t (6.6)	107.5
6	6.60, d (7.2)	142.4
1′		167.4
2′	3.66, m	29.8
3′	5.95, m	127.3
4′	6.04, m	126.1
5′	5.48, t (6.6)	107.5
6′	6.60, d (7.2)	142.4

**Table 2:** Antibacterial activities of compounds **1**–**5** against multi-drug resistant (MDR) strains (MIC, unit: µg/mL).

Strains	1	2	3	4	5
*Staphylococcus aureus*	>12.5	>12.5	>12.5	>12.5	3.12
*Escherichia coli*	>12.5	>12.5	>12.5	>12.5	>12.5
*Enterococcus faecalis*	>12.5	>12.5	>12.5	>12.5	>12.5
*Enterococcus faecium*	>12.5	>12.5	>12.5	>12.5	>12.5
*Salmonella typhimurium*	>12.5	>12.5	>12.5	>12.5	>12.5
*Acinetobacter baumannii*	>12.5	>12.5	>12.5	>12.5	>12.5
*Klebsiella pneumoniae*	>12.5	>12.5	>12.5	>12.5	3.12

## Supplementary Materials

The following are available online at http://www.mdpi.com/1660-3397/16/6/191/s1. Supporting Figures S1−S26 and Tables S1−S4, including HR-ESIMS and NMR spectra of **1**–**5**; plasmids and primer lists.
