# Supplementary material for: Heterologous Expression of a VioA Variant Activates Cryptic Compounds in a Marine-Derived Brevibacterium Strain
Source: Mar Drugs. 2018 Jun 2;16(6):191. doi: 10.3390/md16060191 (PMC6024985; doi:10.3390/md16060191)
Supplement: Supplementary file 1 [file marinedrugs-16-00191-s001.zip › marinedrugs-311250-SI.pdf]

Supporting Information

Heterologous Expression of a VioA Variant Activates Cryptic Compounds in a Marine-derived *Brevibacterium* strain

Xiao Han,[†,⊥] Lukuan Hou,[†,⊥] Jing Hou,[†] Yongyu Zhang,[§] Huayue Li,*[,†,‡] Wenli Li*[,†,‡]

[†]Key Laboratory of Marine Drugs, Ministry of Education, School of Medicine and Pharmacy, Ocean University of China, Qingdao 266003, China

[‡]Key Laboratory of Marine Drugs, Ministry of Education, School of Medicine and Pharmacy, Ocean University of China, Qingdao 266003, China

[§]Qingdao Institute of Biomass Energy and Bioprocess Technology, Chinese Academy of Sciences, Qingdao 266003, China

Table of Contents

Figure S1. Structures of (**A**) violapyrones and (**B**) photopyrones

Figure S2. Gel electrophoresis of PCR products

Figure S3. Culture broth of the wild type and recombinant strains of *Brevibacterium* sp. 7002-073

Figure S4. The HR-ESIMS spectrum of **3**

Figure S5. ^{1}H NMR spectrum of **3** in CD_3OD (600 MHz)

Figure S6. COSY spectrum of **3** in CD_3OD (600 MHz)

Figure S7. HSQC spectrum of **3** in CD_3OD (600 MHz)

Figure S8. HMBC spectrum of **3** in CD_3OD (600 MHz)

Figure S9. NOESY spectrum of **3** in CD_3OD (600 MHz)

Figure S10. The HR-ESIMS spectrum of **1**

Figure S11. ^{1}H NMR spectrum of **1** in CD_3OD (600 MHz)

Figure S12. COSY spectrum of **1** in CD_3OD (600 MHz)

Figure S13. HSQC spectrum of **1** in CD_3OD (600 MHz)

Figure S14. HMBC spectrum of **1** in CD_3OD (600 MHz)

Figure S15. The HR-ESIMS spectrum of **2**

Figure S16. ^{1}H NMR spectrum of **2** in CD_3OD (500 MHz)

Figure S17. The HR-ESIMS spectrum of **4**

Figure S18. ^{1}H NMR spectrum of **4** in CD_3OD (600 MHz)

Figure S19. COSY spectrum of **4** in CD_3OD (600 MHz)

Figure S20. HSQC spectrum of **4** in CD_3OD (600 MHz)

Figure S21. HMBC spectrum of **4** in CD_3OD (600 MHz)

Figure S22. The HR-ESIMS spectrum of **5**

Figure S23. ^{1}H NMR spectrum of **5** in DMSO-d_6 (600 MHz)

Figure S24. COSY spectrum of **5** in DMSO-d_6 (600 MHz)

Figure S25. HSQC spectrum of **5** in DMSO-d_6 (600 MHz)

Figure S26. HMBC spectrum of **5** in DMSO-d_6 (600 MHz)

Table S1. The ^{1}H and ^{13}C NMR chemical shifts of **1** in DMSO-d_6 (600 MHz)

Table S2. The ^{1}H and ^{13}C NMR chemical shifts of **2** in CD_3OD (500 MHz)

Table S3. The ^{1}H and ^{13}C NMR chemical shifts of **4** in CD_3OD (600 MHz)

Table S4. The ^{1}H and ^{13}C NMR chemical shifts of **5** in DMSO-d_6 (600 MHz)

Table S5. The plasmids used in this study

Table S6. Primer pairs used in this study

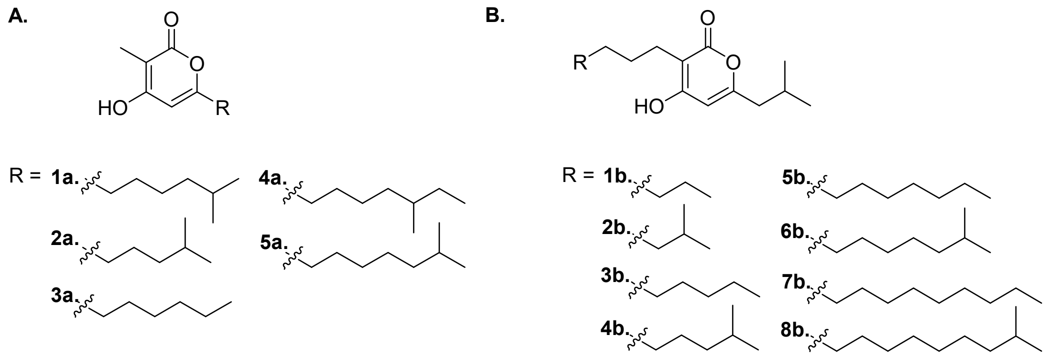

Figure S1. Structures of (**A**) violapyrones and (**B**) photopyrones.

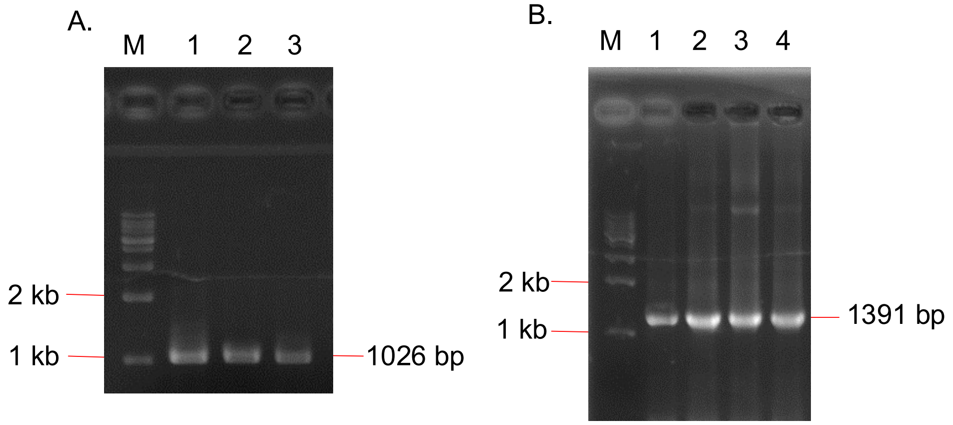

Figure S2. PCR confirmation of 7002-073/pMT3 (A) and 7002-073/pWLI823 (B) strains. (**A**) lane M, 1kb DNA marker; lane 1, pMT3 (positive control); lane 2-3, 7002-073/pMT3 . (**B**) lane M, 1kb DNA marker; lane 1, pWLI823 (positive control); lane 2-4, 7002-073/pWLI823 (lane 2-4).

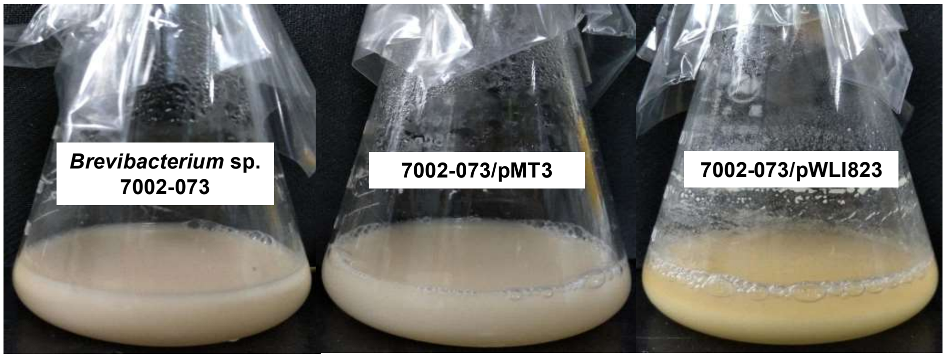

Figure S3. Culture broth of the wild type and recombinant strains of *Brevibacterium* sp. 7002-073.

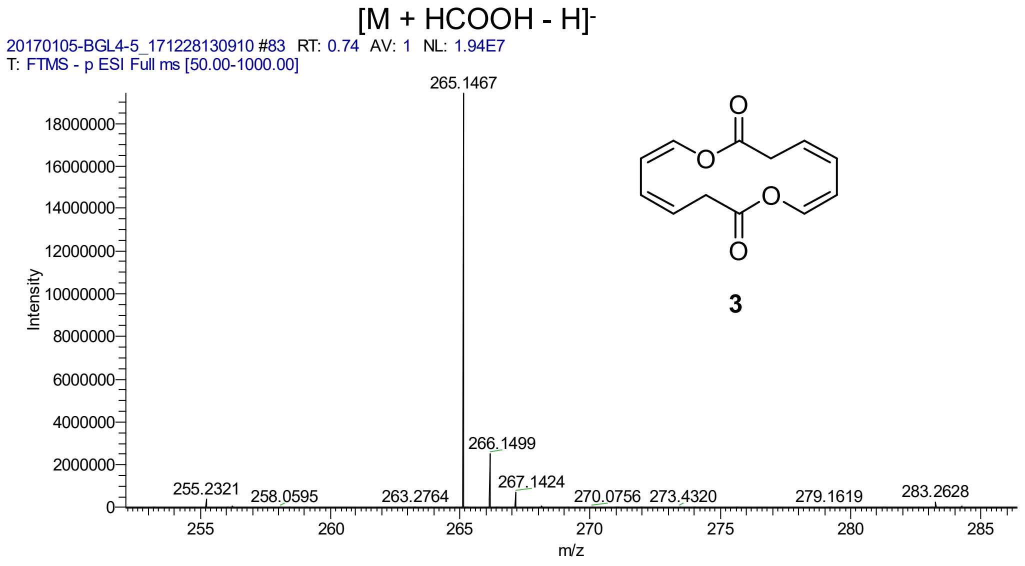

Figure S4. The HR-ESIMS spectrum of **3**

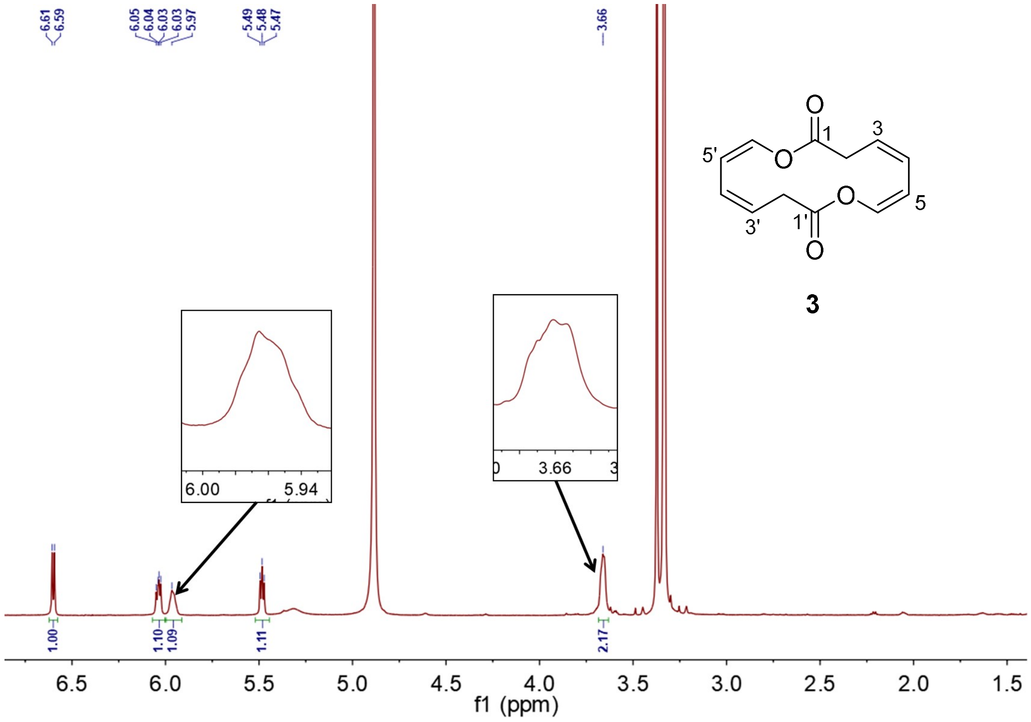

Figure S5. ^{1}H NMR spectrum of **3** in CD_3OD (600 MHz)

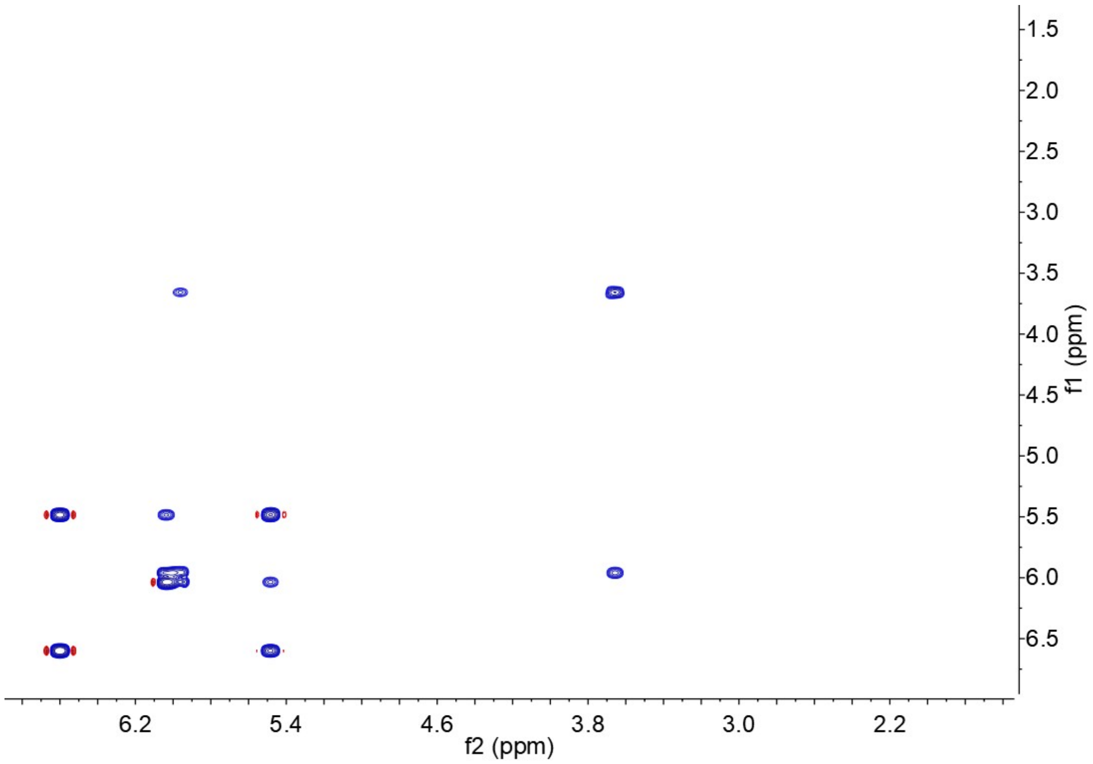

Figure S6. The ^{1}H-^{1}H COSY spectrum of **3** in CD_3OD (600 MHz)

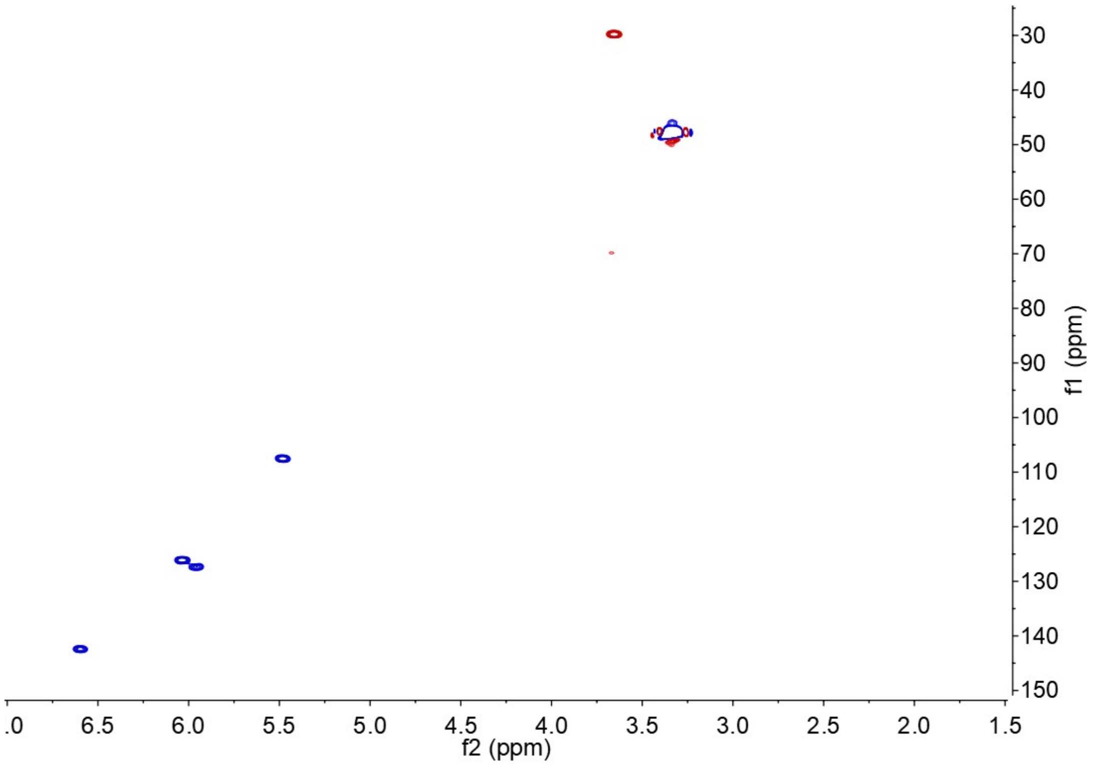

Figure S7. The HSQC spectrum of **3** in CD_3OD (600 MHz)

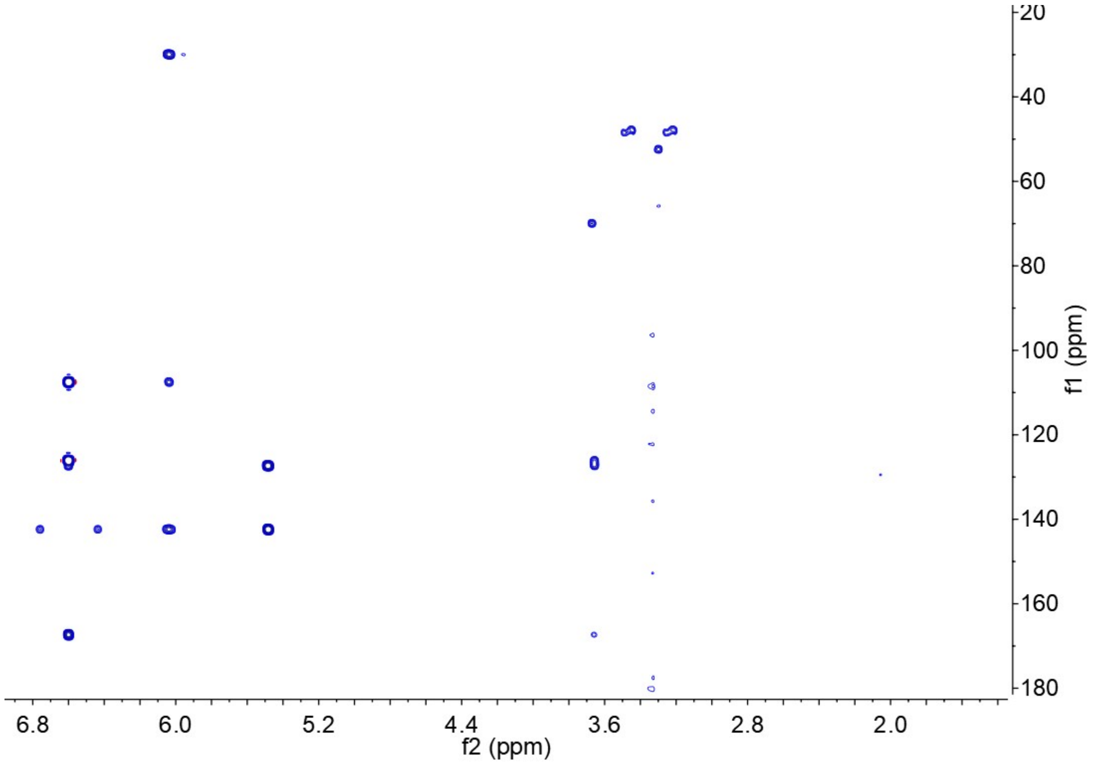

Figure S8. The HMBC spectrum of **3** in CD_3OD (600 MHz)

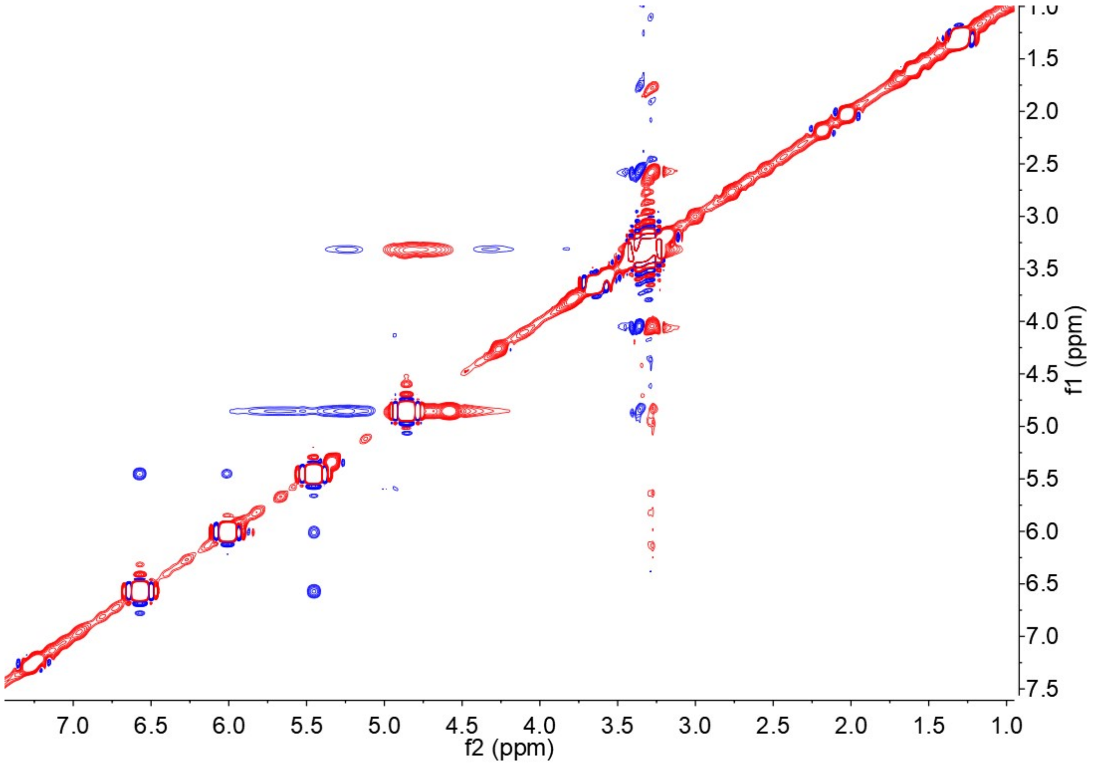

Figure S9. The NOESY spectrum of **3** in CD_3OD (600 MHz)

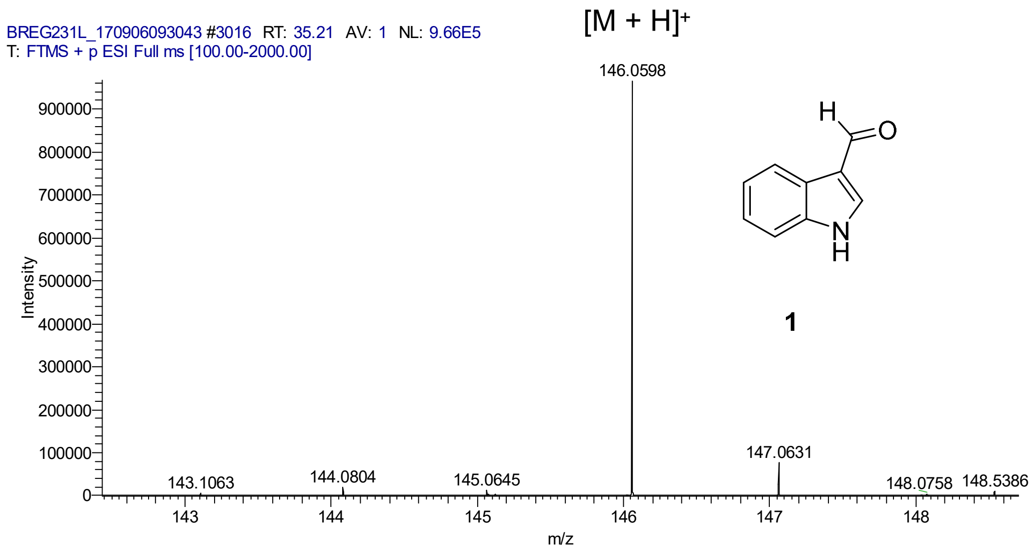

Figure S10. The HR-ESIMS spectrum of **1**

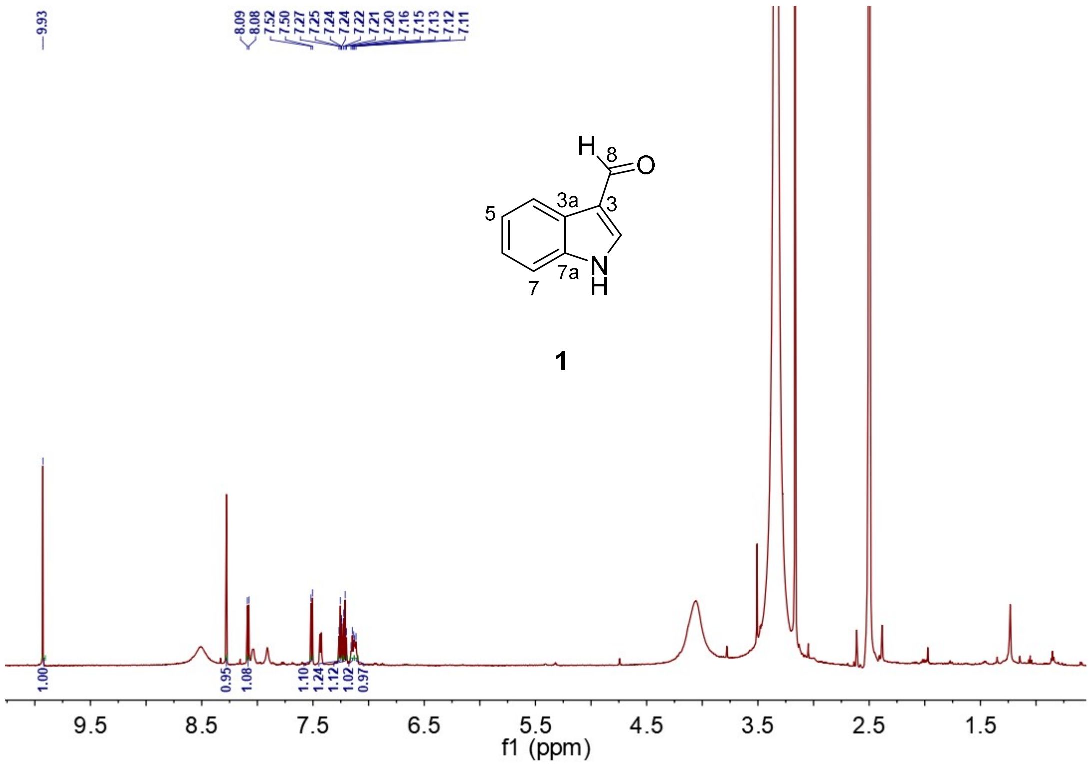

Figure S11. ^{1}H NMR spectrum of **1** in DMSO-d_6 (600 MHz)

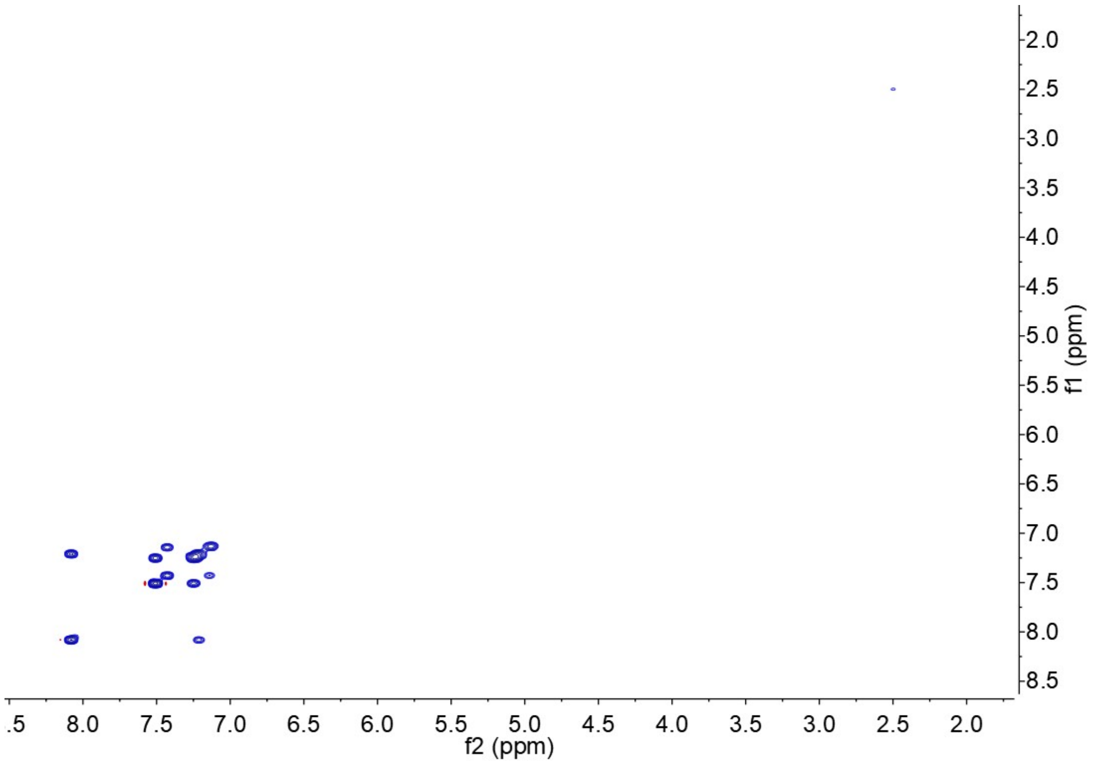

Figure S12. The ^{1}H-^{1}H COSY spectrum of **1** in DMSO-d_6 (600 MHz)

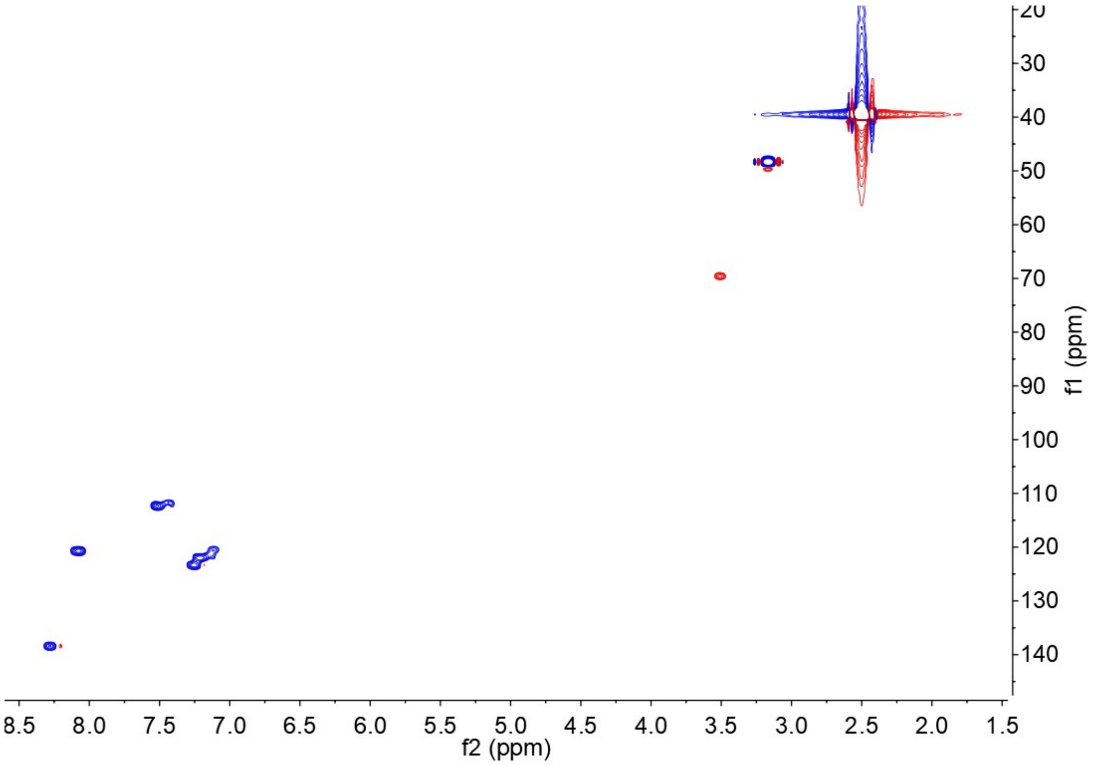

Figure S13. The HSQC spectrum of **1** in DMSO-d_6 (600 MHz)

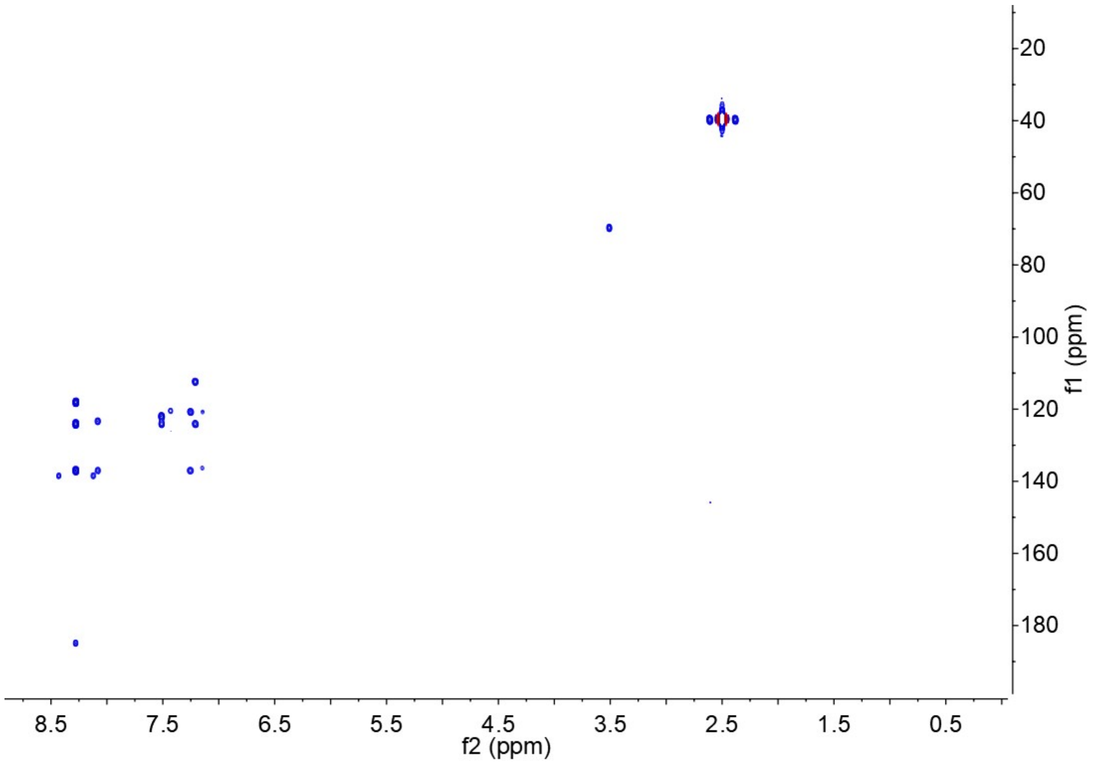

Figure S14. The HMBC spectrum of **1** in DMSO-d_6 (600 MHz)

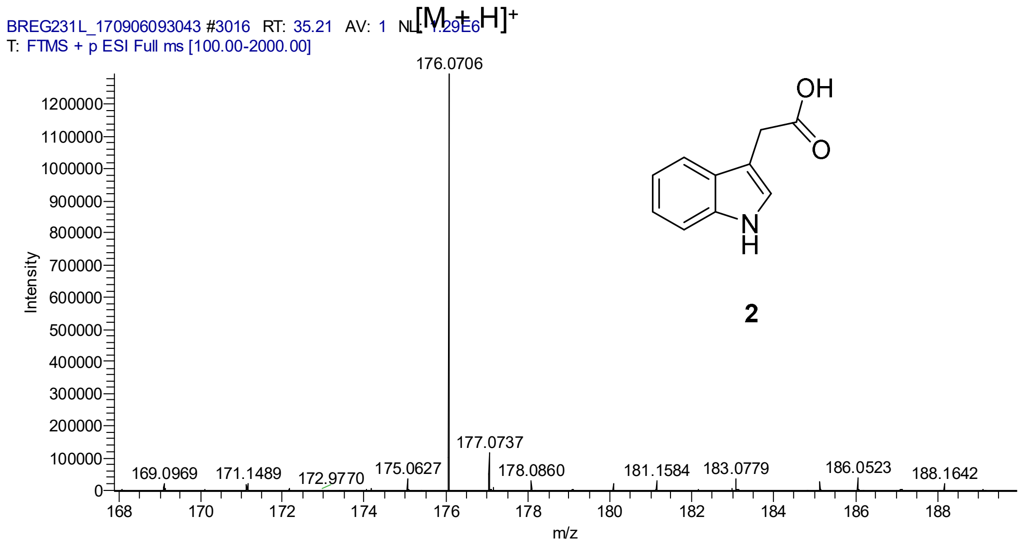

Figure S15. The HR-ESIMS spectrum of **2**

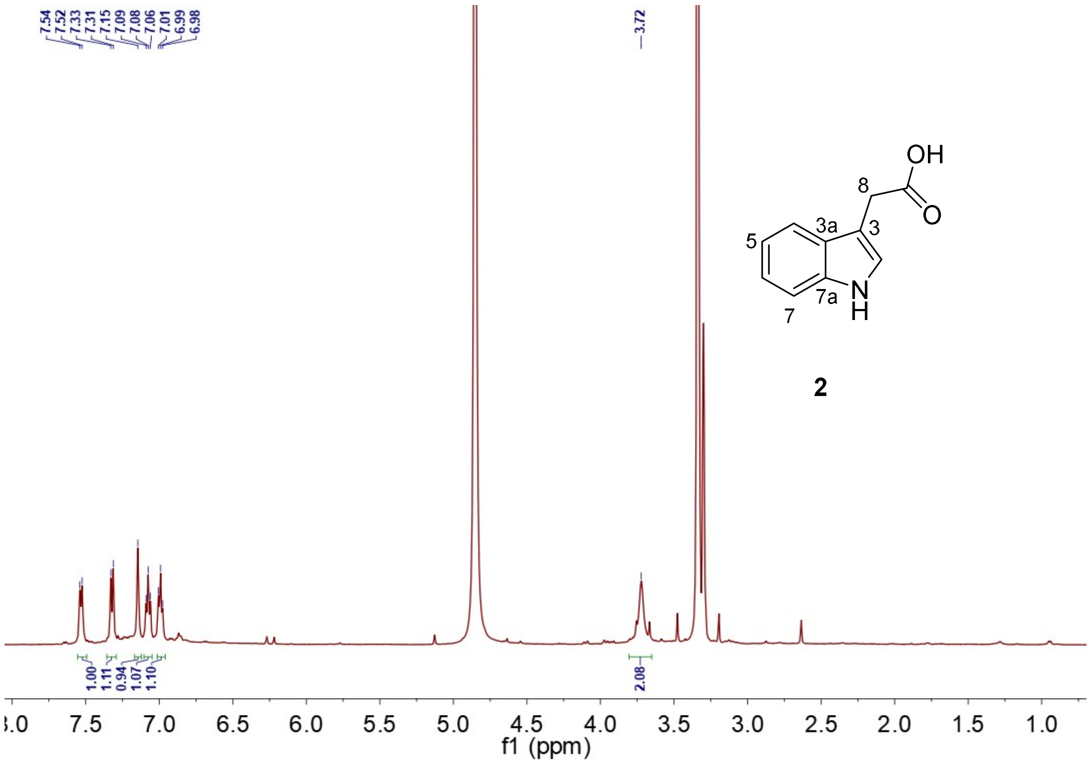

Figure S16. ^{1}H NMR spectrum of **2** in CD_3OD (500 MHz)

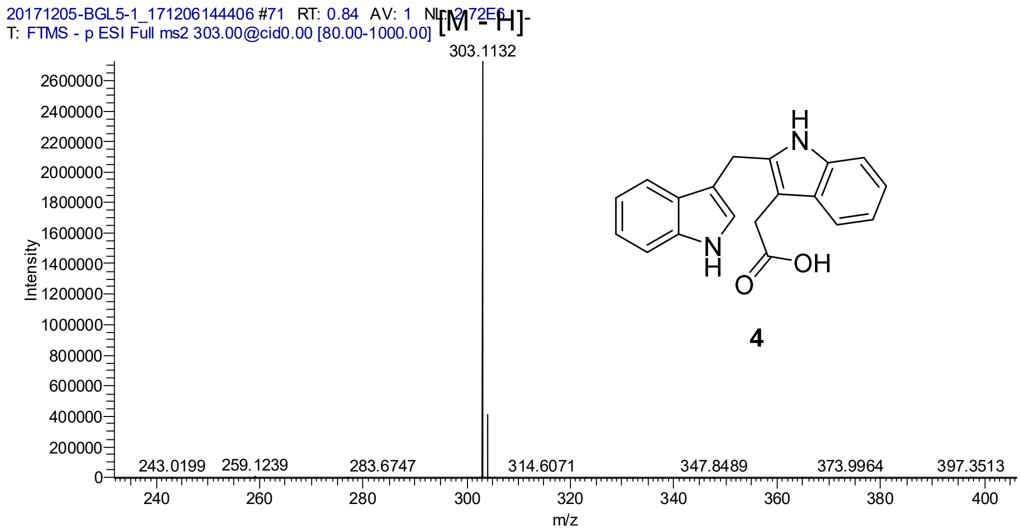

Figure S17. The HR-ESIMS spectrum of **4**

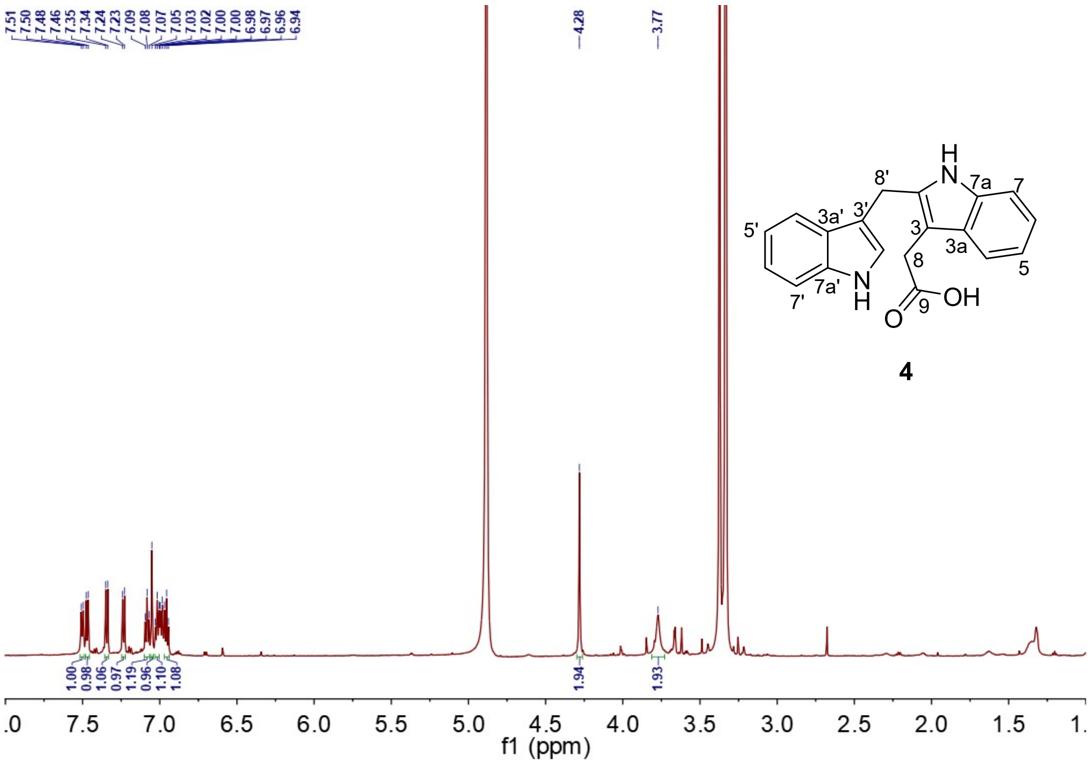

Figure S18. ^{1}H NMR spectrum of **4** in CD_3OD (600 MHz)

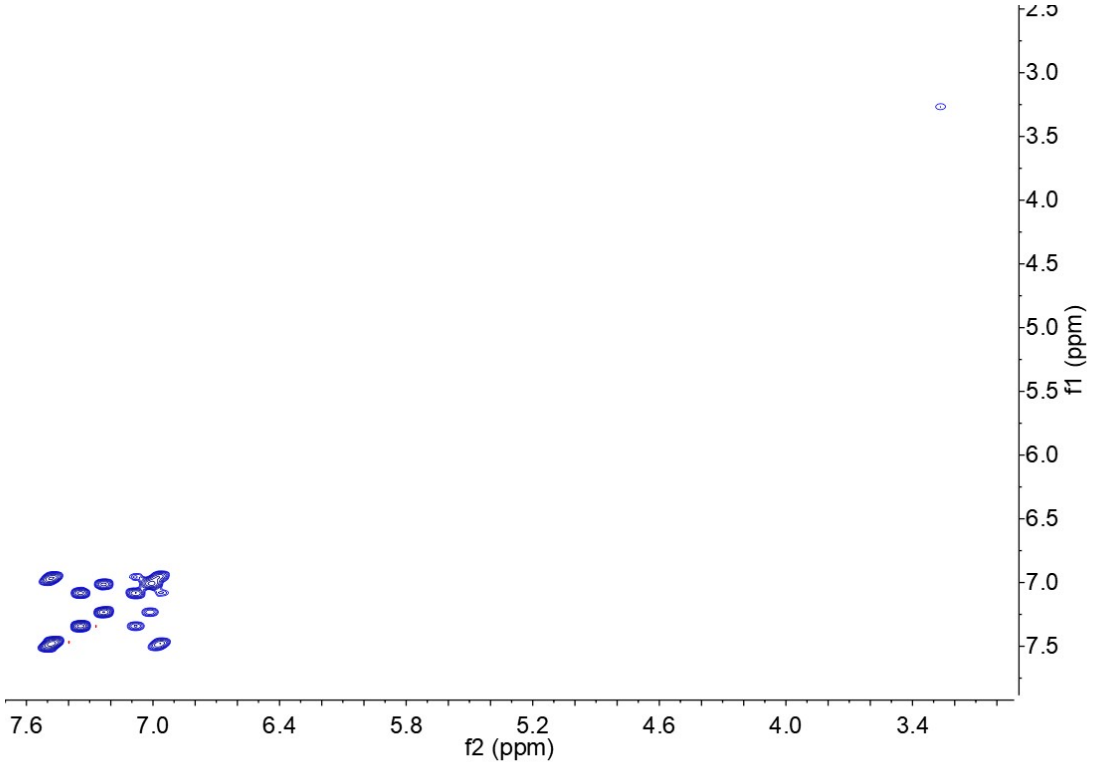

Figuer S19. The ^{1}H-^{1}H COSY spectrum of **4** in CD_3OD (600 MHz)

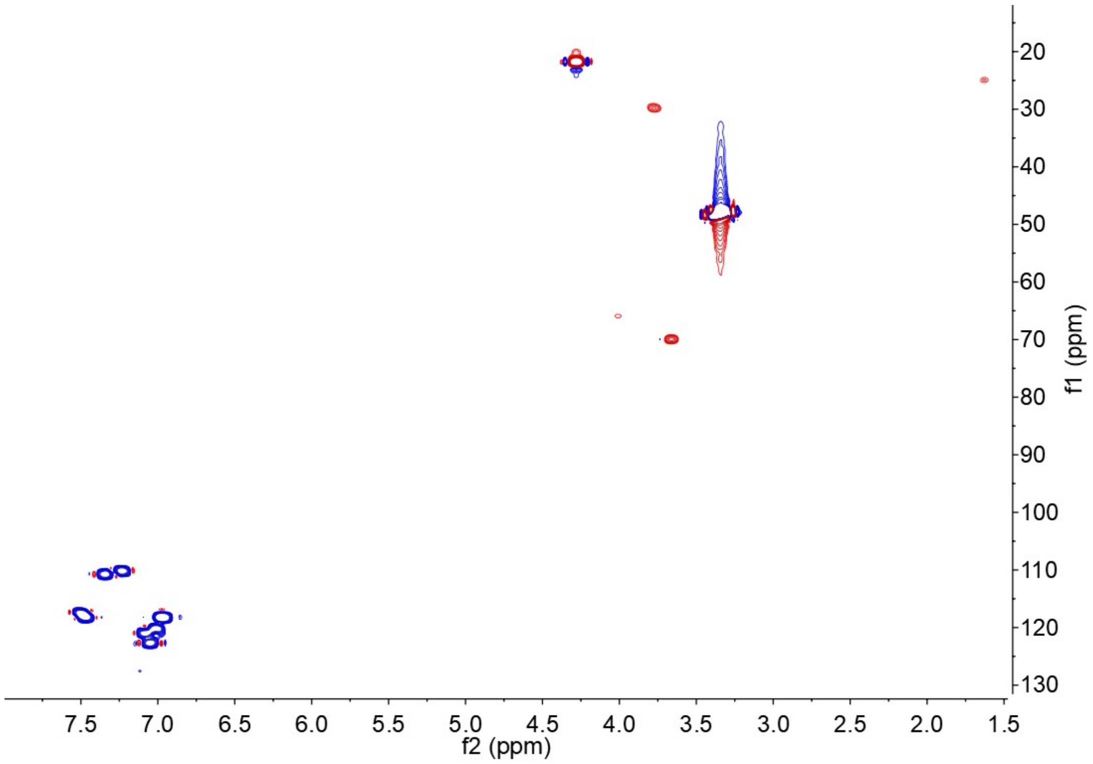

Figure S20. The HSQC spectrum of **4** in CD_3OD (600 MHz)

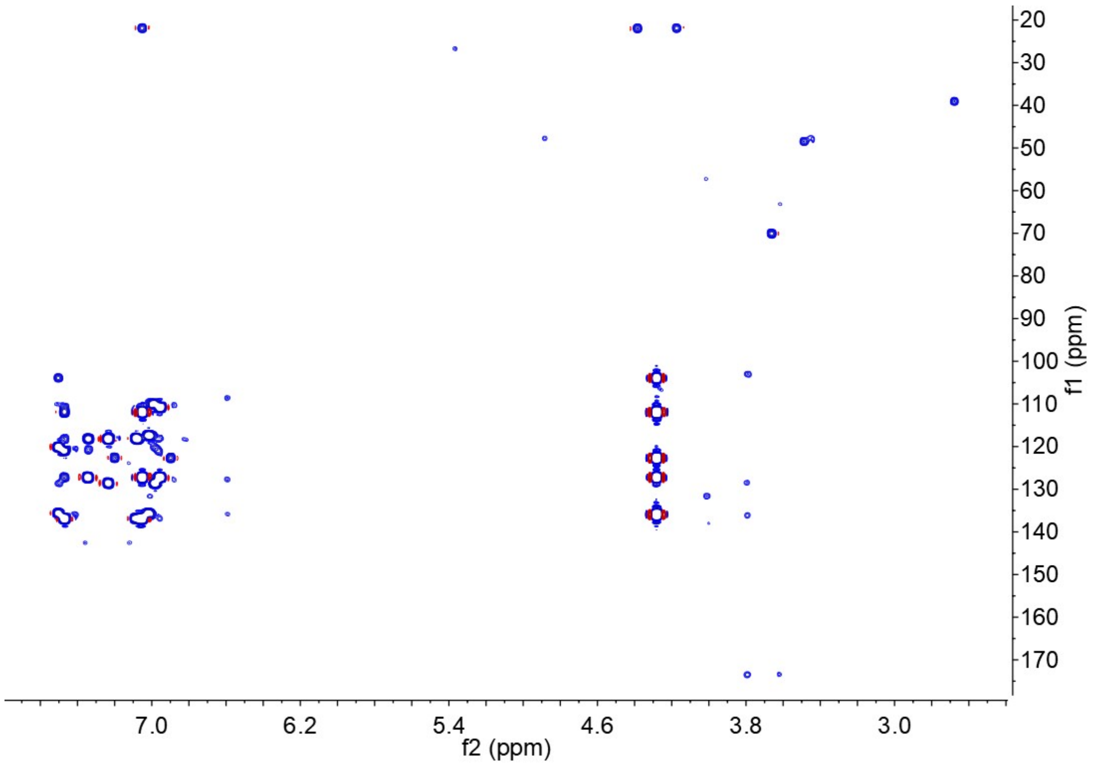

Figure S21. The HMBC spectrum of **4** in CD_3OD (600 MHz)

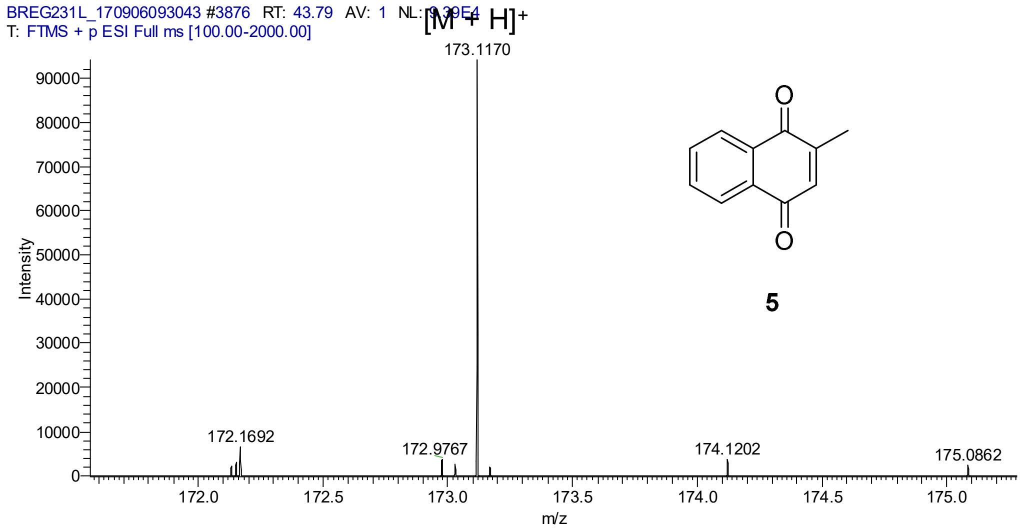

Figure S22. The HR-ESIMS spectrum of **5**

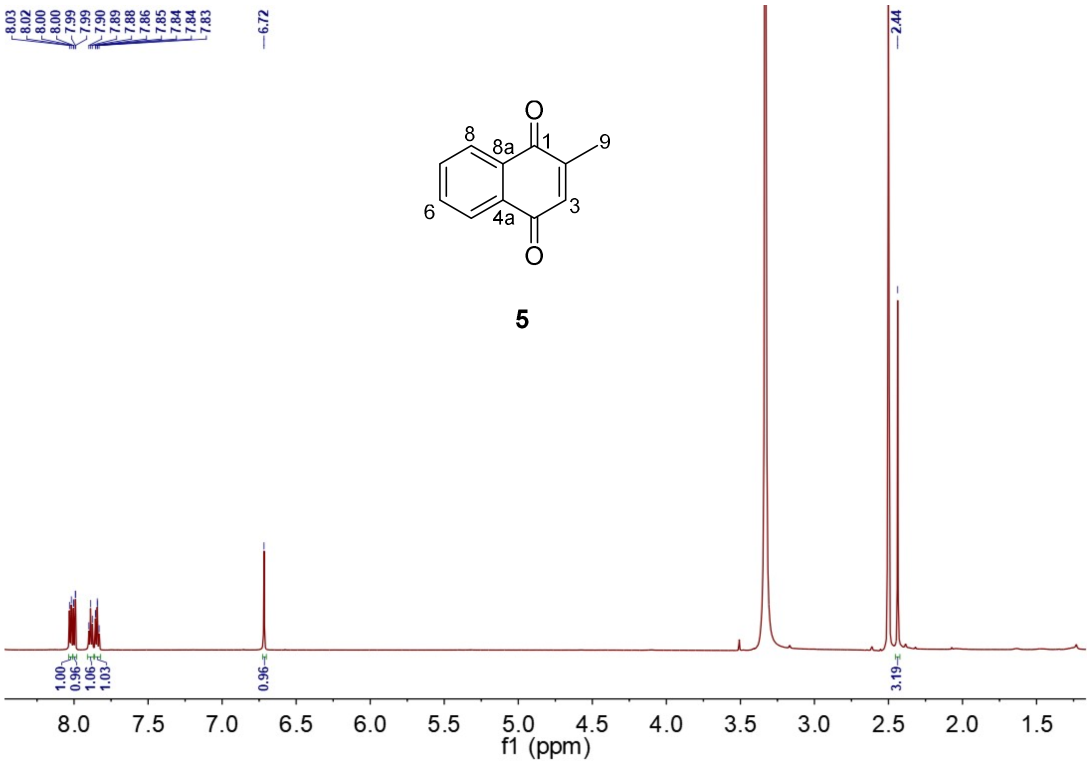

Figure S23. ^{1}H NMR spectrum of **5** in DMSO-d_6 (600 MHz)

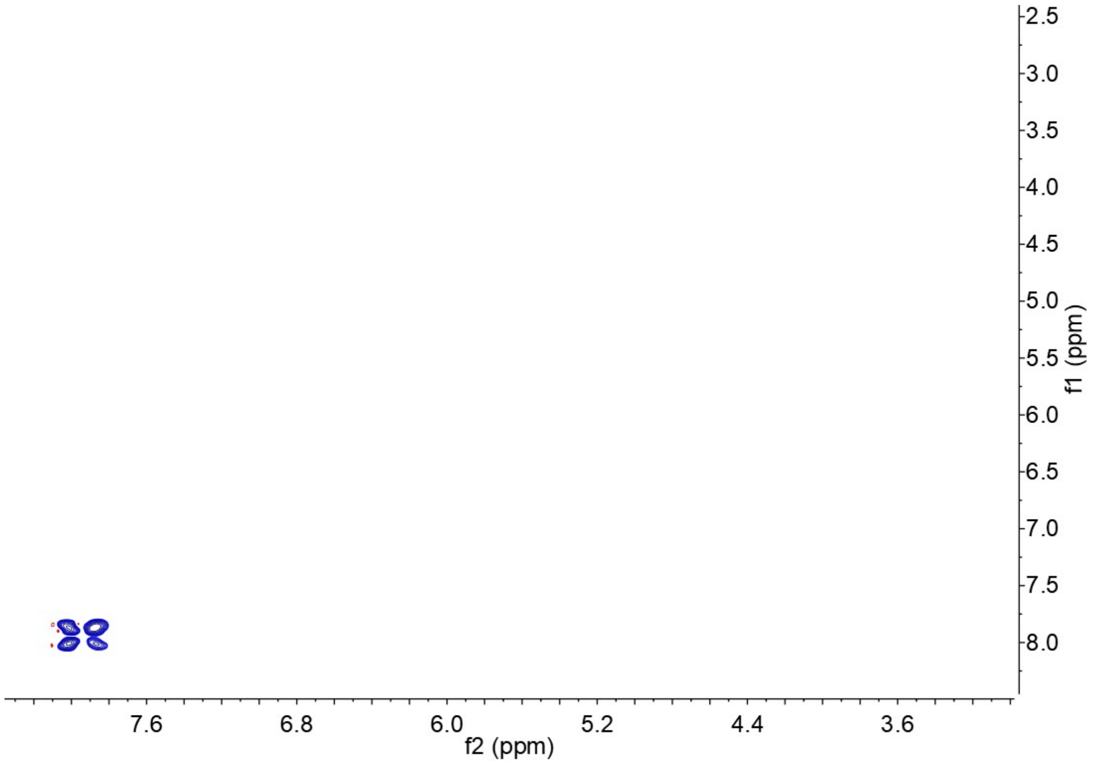

Figure S24. The ^{1}H-^{1}H COSY spectrum of **5** in DMSO-d_6 (600 MHz)

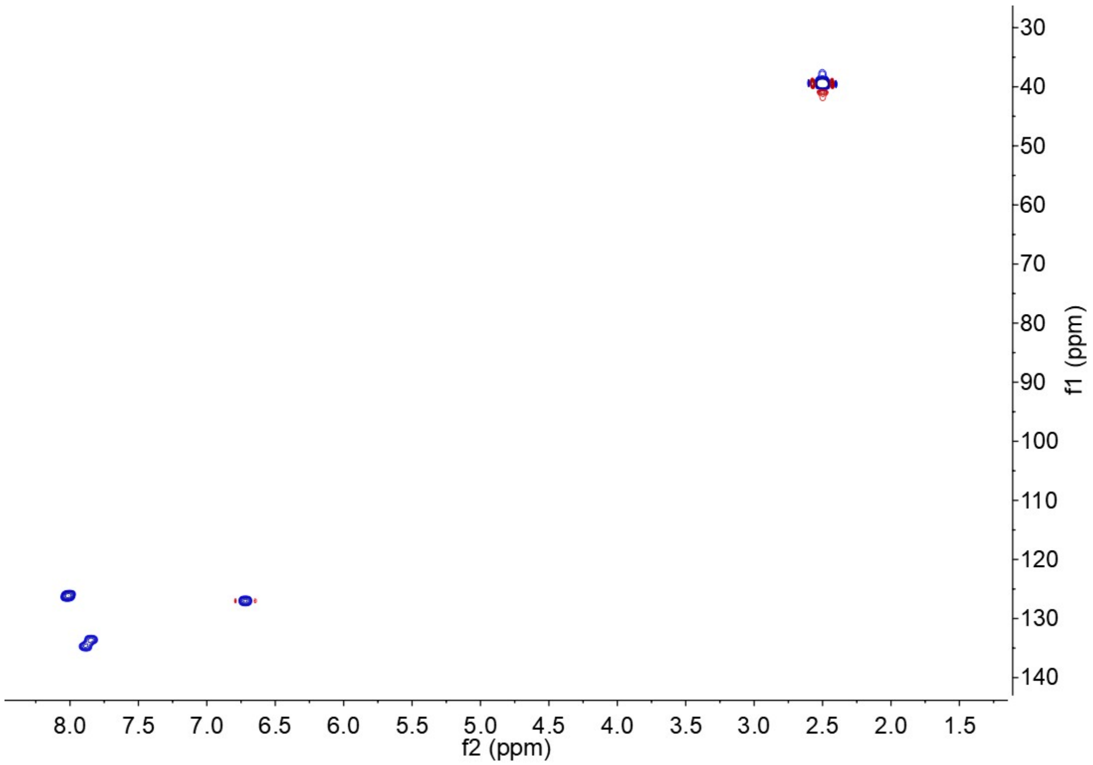

Figure S25. The HSQC spectrum of **5** in DMSO-d_6 (600 MHz)

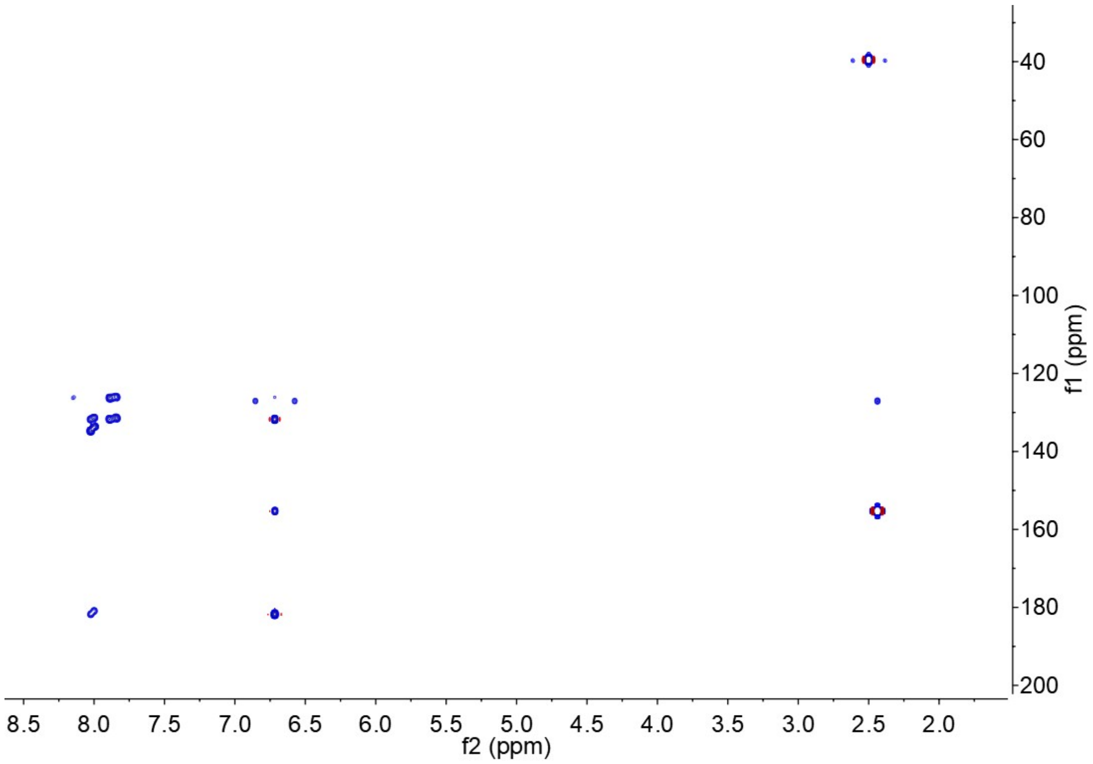

Figure S26. The HMBC spectrum of **5** in DMSO-d_6 (600 MHz)

Table S1. The ^{1}H and ^{13}C NMR chemical shifts of **1** in DMSO-d_6 (600 MHz)

Position	**1**	
2	8.28 (1H, s)	138.4
3		118.1
4	8.08 (1H, d, 7.2)	120.7
5	7.21 (1H, m)	122.0
6	7.25 (1H, m)	123.3
7	7.52 (1H, d, 8.4)	112.3
8	9.93 (1H, s)	184.9
3a		124.1
7a		137.1

Table S2. The ^{1}H and ^{13}C NMR chemical shifts of **2** in CD_3OD (500 MHz)

Position	**2**	
2	7.15 (1H, s)	123.2
3		108.0
4	7.53 (1H, d, 7.5)	118.1
5	6.99 (1H, t, 7.0)	118.3
6	7.08 (1H, t, 7.0)	121.0
7	7.32 (1H, d, 7.5)	110.8
8	3.72 (2H, s)	30.6
9		173.3
3a		127.3
7a		136.5

Table S3. The ^{1}H and ^{13}C NMR chemical shifts of **4** in CD_3OD (600 MHz)

Position	**4**	
2		135.9
3		103.9
4	7.50 (1H, d, 7.8)	117.4
5	6.99 (1H, m)	118.2
6	7.01 (1H, m)	120.1
7	7.23 (1H, d, 7.2)	110.2
8	3.77 (2H, s)	29.8
9		173.5
2'	7.05 (1H, s)	122.7
3'		112.7
4'	7.47 (1H, d, 8.4)	118.3
5'	6.95 (1H, m)	118.2
6'	7.08 (1H, t, 7.8)	121.0
7'	7.34 (1H, d, 7.8)	110.7
8'	4.28 (2H, s)	21.8
3a		128.6
7a		135.7
3a'		127.2
7a'		136.9

Table S4. The ^{1}H and ^{13}C NMR chemical shifts of **5** in DMSO-d_6 (600 MHz)

Position	**5**	
1		181.8
2		155.3
3	6.72 (1H, s)	127.0
4		180.9
5	8.00 (1H, d, 7.8)	126.1
6	7.89 (1H, t, 6.6)	134.7
7	7.85 (1H, t, 6.6)	133.6
8	8.02 (1H, d, 7.8)	126.4
9	2.44 (3H, s)	12.7
4a		131.8
8a		131.4

Table S5. The plasmids used in this study.

Plasmids	Description	Reference or source
pWLI806	pMT3, Integrative vector, derivative of pSET152	[1]
pWLI823	pWLI807 derivative where G231 in VioA was mutated to leucine	unpublished data

Table S6. Primer pairs used in this study

gene	Primer pairs used for PCR confirmation of the strains (5'-3')
thio	*thio*CF: GGCCCGATCAAGGCGAATAC
	*thio*CR: CCGAGGAACAGAGGCGCTTA
vioA	*vioA*CF:CGCACCCCCTGGTCAACGCG
	*vioA*CR:TCACGCCGCCCAGACCCCAC

Reference

(1) Huang, H.; Hou, L.; Li, H.; Qiu, Y.; Ju, J.; Li, W. *Microb. Cell Fact.* **2016**, 15, 116.
